# Supplementary material for: Isolation, Identification and Function of Pichia anomala AR2016 and Its Effects on the Growth and Health of Weaned Pigs
Source: Animals (Basel). 2021 Apr 20;11(4):1179. doi: 10.3390/ani11041179 (PMC8074749; doi:10.3390/ani11041179)
Supplement: Supplementary file 1 [file animals-11-01179-s001.zip › animals-1159967-supplementary.pdf]

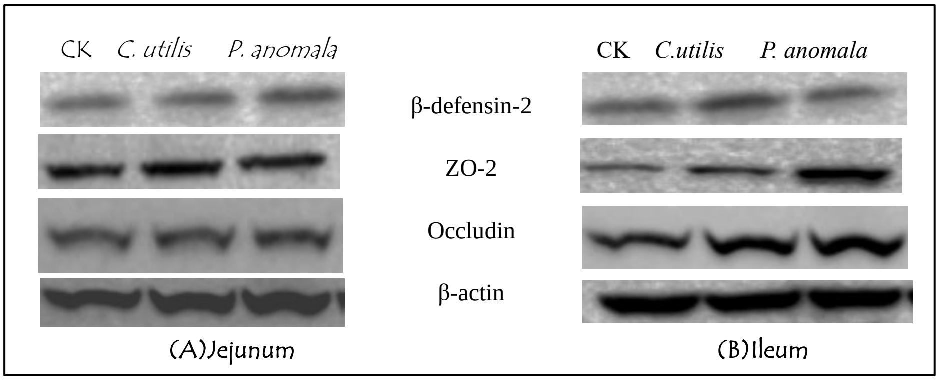
CK
C. utilis
P. anomala
CK
C.utilis
P. anomala
β-defensin-2
ZO-2
Occludin
β-actin
(A)Jejunum
(B)Ileum

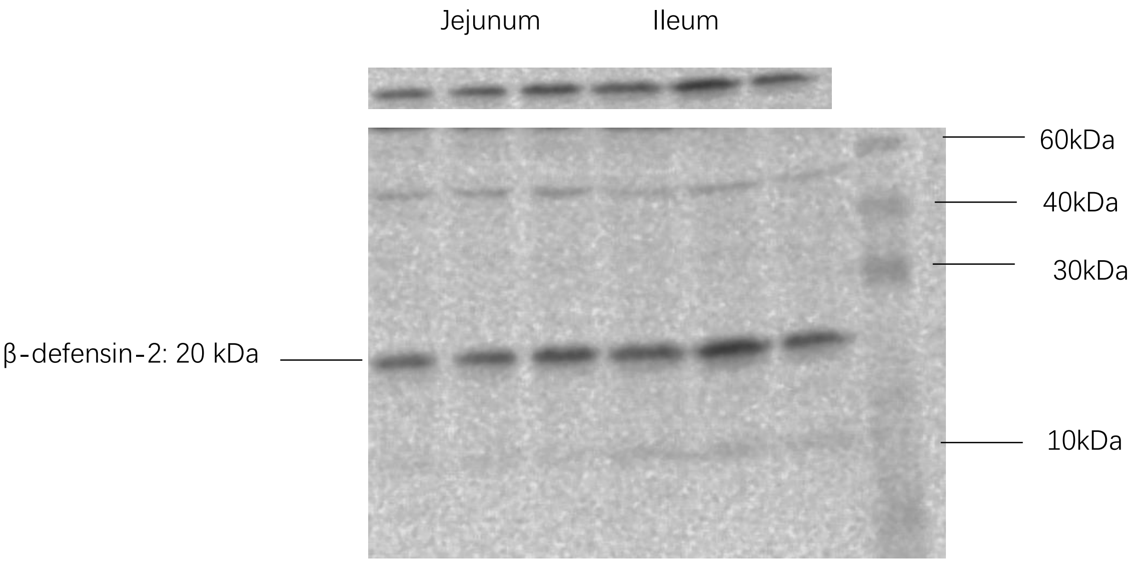
Jejunum
Ileum
60kDa
40kDa
30kDa
β-defensin-2: 20 kDa
10kDa

Jejunum β-actin

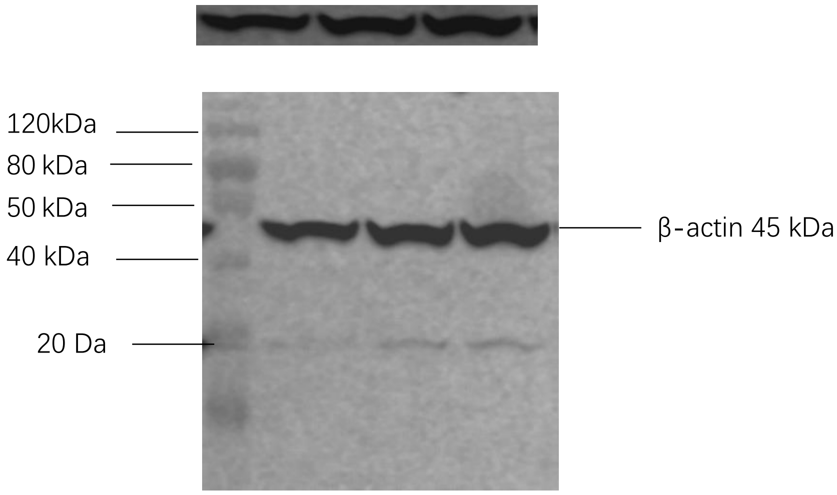

Ileum β-actin

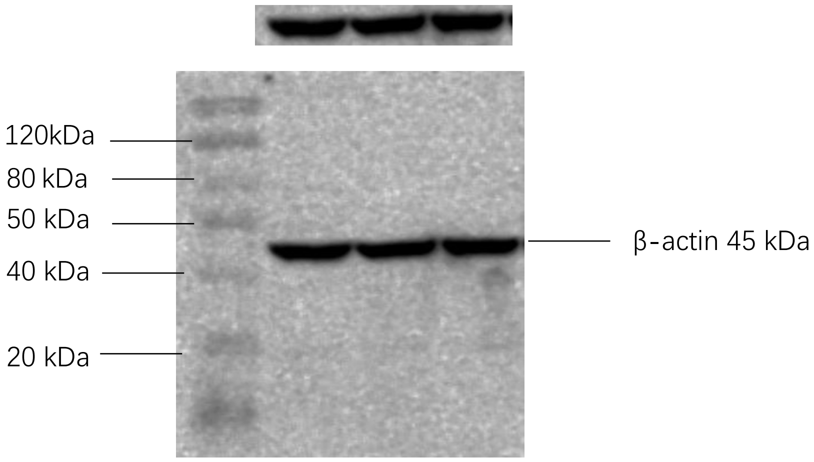

Jejunum Occludin 57 kDa

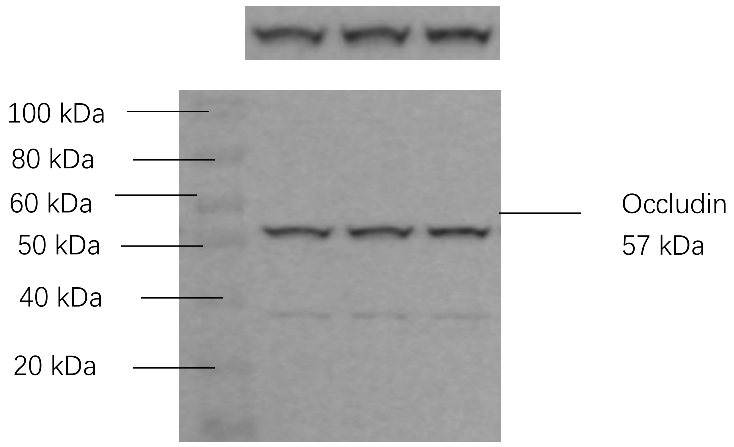

Ileum Occludin 57 kDa

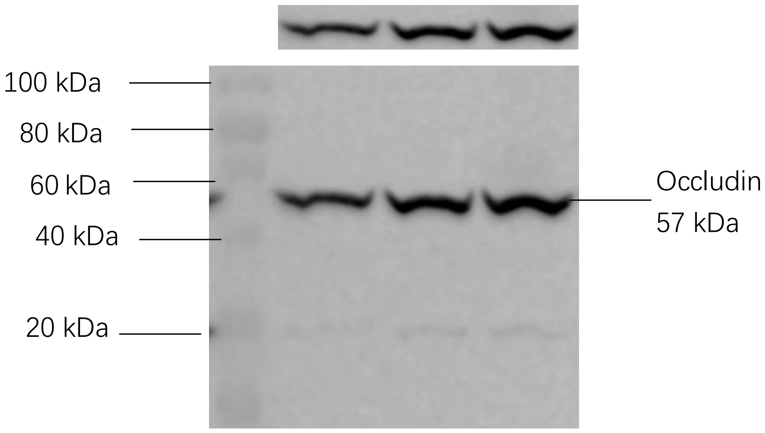

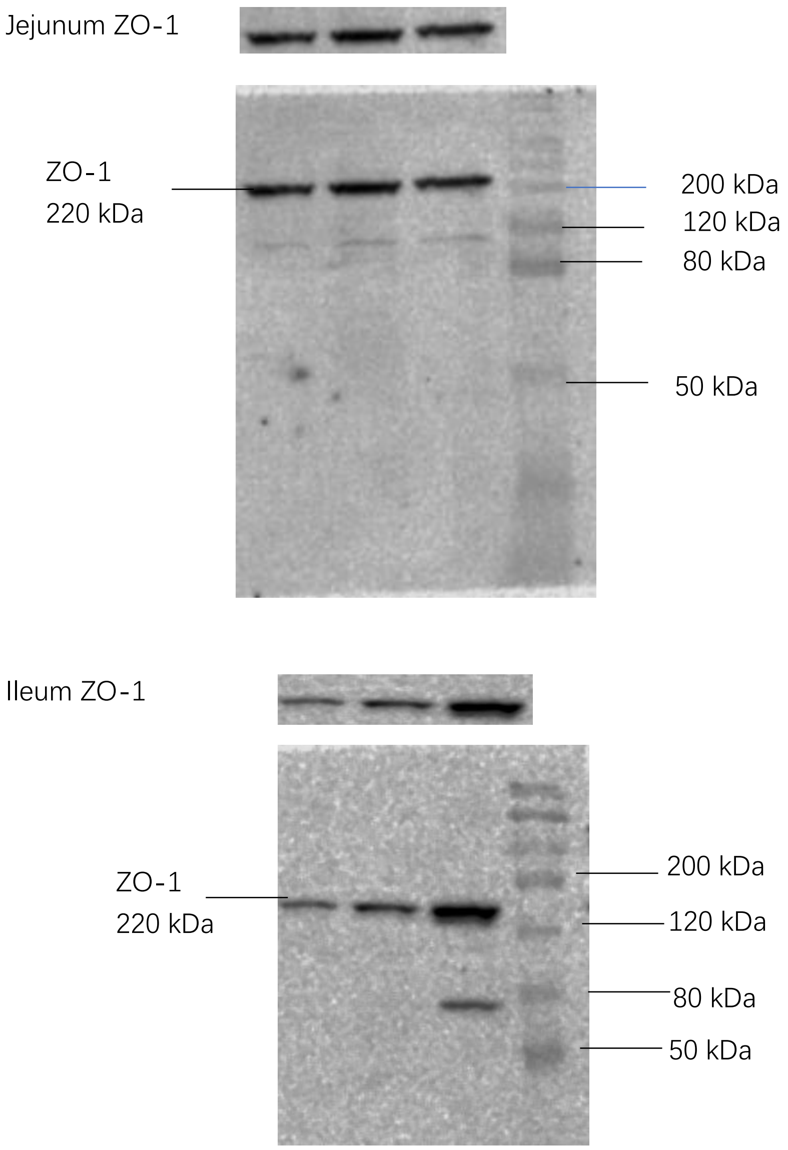
Jejunum ZO-1
ZO-1
220 kDa
200 kDa
120 kDa
80 kDa
50 kDa
Ileum ZO-1
ZO-1
220 kDa
200 kDa
120 kDa
80 kDa
50 kDa
